# Factors Associated With Worsened Mental Health of Health Care Workers in Canada During the COVID-19 Pandemic: Cross-Sectional Survey Study

**DOI:** 10.2196/50064

**Published:** 2024-02-15

**Authors:** Ali AbdulHussein, Zahid Ahmad Butt, Stanko Dimitrov, Brian Cozzarin

**Affiliations:** 1 Department of Management Science Faculty of Engineering University of Waterloo Waterloo, ON Canada; 2 School of Public Health Sciences Faculty of Health University of Waterloo Waterloo, ON Canada

**Keywords:** health care workers, COVID-19, mental health, demographic factors, occupational factors, access to PPE, pandemic, health care system, psychological trauma, psychological, trauma, respirators, eye protection, face shields, support

## Abstract

**Background:**

Health care workers (HCWs) in Canada have endured difficult conditions during the COVID-19 pandemic. Many worked long hours while attending to patients in a contagious environment. This introduced an additional burden that may have contributed to worsened mental health conditions.

**Objective:**

In this study, we examine the factors associated with worsened mental health conditions of HCWs as compared to before the start of the pandemic.

**Methods:**

We use data from a survey of HCWs by Statistics Canada. A regression model is used to estimate the odds ratios (ORs) of worsened mental health after the start of the pandemic. The estimated odds ratio (OR) is associated with different independent variables that include demographics (age, sex, immigration status, and geographic area), occupational factors (work status, occupational group, and exposure category), and different access levels to personal protective equipment (PPE).

**Results:**

Of 18,139 eligible participants surveyed, 13,990 (77.1%) provided valid responses. We found that HCWs younger than 35 years old were more likely (OR 1.14, 95% CI 1.03-1.27; *P*=.01) to exhibit worsened mental health as compared to the reference group (35-44 years old). As for sex, male HCWs were less likely (OR 0.76, 95% CI 0.67-0.86; *P*<.001) to exhibit worsened mental health as compared to female HCWs. Immigrant HCWs were also less likely (OR 0.57, 95% CI 0.51-0.64; *P*<.001) to exhibit worsened mental health as compared to nonimmigrant HCWs. Further, HCWs working in Alberta had the highest likelihood of exhibiting worsened mental health as compared to HCWs working elsewhere (Atlantic provinces, Quebec, Manitoba, Saskatchewan, Ontario, British Columbia, and Northern Territories). Frontline workers were more likely (OR 1.26, 95% CI 1.16-1.38; *P*<.001) to exhibit worsened mental health than nonfrontline HCWs. Part-time HCWs were less likely (OR 0.85, 95% CI 0.76-0.93; *P*<.001) to exhibit worsened mental health than full-time HCWs. HCWs who reported encountering COVID-19 cases were more likely (OR 1.55, 95% CI 1.41-1.70; *P*<.001) to exhibit worsened mental health as compared to HCWs who reported no contact with the disease. As for PPE, HCWs who never had access to respirators, eye protection, and face shields are more likely to exhibit worsened mental health by 1.31 (95% CI 1.07-1.62; *P*<.001), 1.51 (95% CI 1.17-1.96; *P*<.001), and 1.41 (95% CI 1.05-1.92; *P*=.02) than those who always had access to the same PPE, respectively.

**Conclusions:**

Different HCW groups experienced the pandemic differently based on their demographic and occupational backgrounds as well as access to PPE. Such findings are important to stakeholders involved in the planning of personalized support programs and aid mental health mitigation in future crises. Certain groups require more attention.

## Introduction

On March 11, 2020, the World Health Organization declared COVID-19 a global pandemic. The pandemic resulted in devastating health impacts on populations and a crisis within the health care system [1]. This health care system was tasked to handle the unprecedented inflow of patients. Functions within the system that were impacted include emergency departments, intensive care units, physician services, and long-term care units [2]. Health care workers (HCWs) of different occupational groups battled the pandemic. Overwhelmed hospitals in Canada canceled less urgent surgeries by up to 80% by June 2020 [3]. These patterns shifted the workload on HCWs and had an impact on the overall health care system. These conditions not only demanded more hospital capacity but also put an overwhelming strain on HCWs [4]. In addition to the operational pressure, HCWs also suffered from the lack of personal protection equipment (PPE), especially at the beginning of the pandemic [5]. HCWs were at the frontline in battling this pandemic. This battle has put pressure on their mental health conditions [6-8].

In this paper, we assess the various factors associated with worsened HCWs’ mental health conditions as compared to before the start of the pandemic. Although experiencing mental health conditions may be a daily occurrence for some HCWs, the duration and severity during the pandemic were different. HCWs were also at a higher risk of infection, adding to the risk of further mental health conditions [9]. In the past, HCWs have experienced mental health problems during other outbreaks, including the Middle East respiratory syndrome and the severe acute respiratory syndrome [10]. Such conditions have been studied in the literature [11]. In this study, we use a recent data set by Statistics Canada from a national cross-sectional survey that was conducted in the fall of 2020 to assess the impact of COVID-19 on HCWs. Unlike work in the literature, we comprehensively assess the impact of demographic and occupational factors as well as the availability of PPE on HCWs’ mental health conditions [9,12,13].

In the global literature, authors assessed the mental health conditions of HCWs during such outbreaks globally [14-16]. The impact of demographic, social, and occupational factors was reportedly linked to various mental health conditions. Researchers also assessed the prevalence of stress, anxiety, and other psychological well-being indicators of HCWs in Oman during the pandemic [12]. The focus was on young female HCWs who encountered confirmed or suspected COVID-19 cases during their work. Another study in Turkey examined the relationship between the perceived risk of infection and the mental health conditions of HCWs during the COVID-19 pandemic [13]. In addition, a study in the Chinese province of Hubei was conducted early in the pandemic to assess the psychological impact of the pandemic on the frontline medical staff [9]. The study measured the association of factors including professional group, age, and sex factors with work stress.

Our study is among the first to highlight the association of diverse demographic and occupational factors with the mental health condition of HCWs during the pandemic in Canada. We also considered the role of access to PPE on HCWs’ mental health conditions. Unlike existing literature, we studied the individual impact of each of the following factors on the mental health of HCWs while holding the rest constant. Demographic factors include age group, sex, province of the workplace, and immigration status. Occupational factors include work status, frontline category, and exposure to confirmed or suspected COVID-19 cases. Finally, we also considered access to several PPE. The findings of the study will be of prime importance to key stakeholders, including mental health support program planners, health care policy makers, HCWs themselves, and researchers in the area. The goal of this paper is to understand which factors were associated with worsened mental health conditions in HCWs after the start of the pandemic.

## Methods

### Data Sources, Study Procedure, and Participants

We used a data set from a recent cross-sectional survey by Statistics Canada on the impacts of COVID-19 on HCWs. Unlike other traditional Statistics Canada surveys, a random selection of participants was not used. Instead, Statistics Canada sent an email invitation to HCWs across Canada. Then, a snowball sampling procedure was used. The invitation included a link to a web-based survey that was available through Statistics Canada’s web page. Accordingly, 18,139 responses were collected between November 24 and December 13, 2020, across 7 provincial and territorial regions in Canada. No data were collected for this survey beyond these dates. Only responses by HCWs were included in the data set. The responses were completely anonymized by Statistics Canada.

The questionnaire asked HCWs for information related to the job environment, demographics, geography, and information on access to PPE as background information. Adaptive questioning was used. Our study was limited to these 3 factors categories only: demographics, occupational, and access to PPE based on the available data from Statistics Canada. In assessing mental health well-being, the survey asked HCWs: compared to before the COVID-19 pandemic, how would you say your mental health is now? HCWs self-reported their perceived mental health on a 5-point Likert scale: much better now, somewhat better now, about the same, somewhat worse now, much worse now.

### Statistical Analysis Strategy

**Figure 1:**
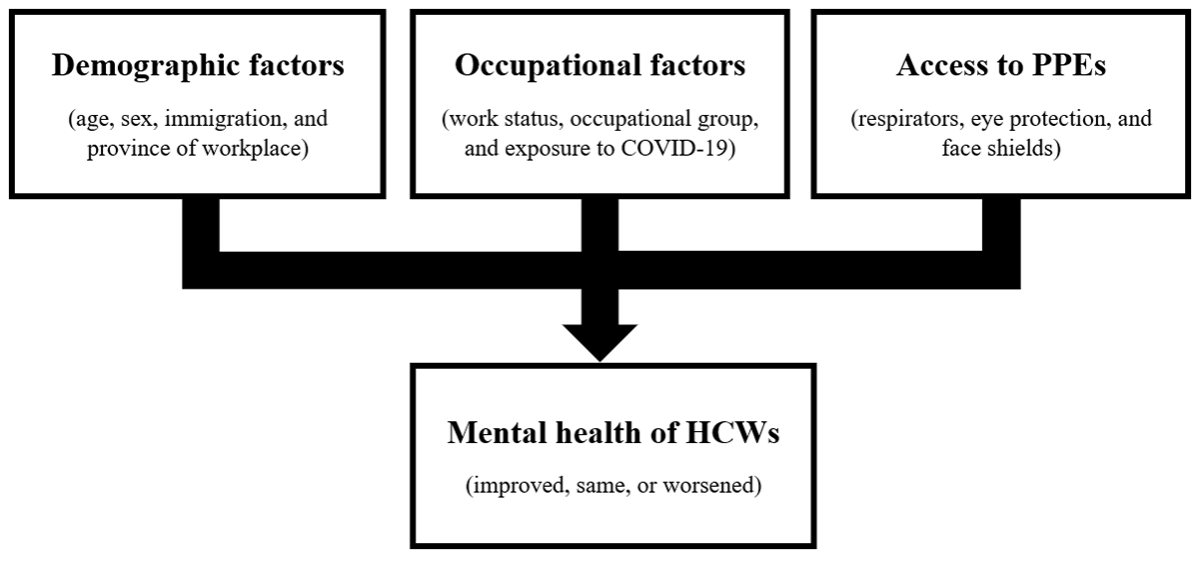
Factors associated with the mental health of HCWs. HCW: health care worker; PPE: personal protective equipment.

Our study uniquely studied the association of various interesting factors, as depicted in Figure 1, with HCW mental health conditions as compared to before the start of the pandemic. To consider each factor separately, we used a multivariate ordinal logistic regression [17,18]. The model is defined in Multimedia Appendix 1. The dependent variable is the state of mental health of the respondents. We reduced the mental health state categories from the 5 mentioned above to 3 (improved, same, or worsened) to yield statistically significant model estimates. The independent variables include demographics such as age, sex, immigration status, and the province of the workplace. Occupational variables include work status, occupational group, and exposure to COVID-19 cases. The model also estimated the association of HCWs’ access to a variety of PPE, such as respirators, eye protection, and face shields. Access to PPE is also reported by the HCW on a 5-point Likert scale, which we reduced to 3 categories (always available, sometimes available, or never available) to yield statistically significant model estimates. All responses are self-reported. Analyses were conducted in RStudio (version 1.4.1717; Posit, PBC).

### Ethical Considerations

Data collected by Statistics Canada were reviewed based on the following principles: privacy, security, transparency, accountability, trust, sustainability, data quality, and fairness, as well as well vetted to be harmless to participants or the public. All ethical approvals were considered by Statistics Canada. No further ethical approval to use these data was required by the authors and the survey is made available to the public. This study is exempt from further ethical approval. Furthermore, the authors also did not have access to information related to the development and testing of the survey. The authors are also not aware of any compensation provided to survey participants.

## Results

### Participant Profile

From the full data set of 18,139 responses, we considered nonduplicate responses that provided valid answers to all questions of interest to this study (N=13,990). The remaining responses contained missing or invalid responses. For the considered population, Table 1 outlines the number of observations and percentages for different demographic factors such as age group, sex, and immigration status. The number of observations and percentages were also calculated for occupational factors including the province of the primary workplace, work status (full-time vs part-time), frontline work status, occupational group, and exposure to confirmed or suspected COVID-19 cases. Additionally, the last 3 rows of Table 1 outline PPE access levels for 3 different PPE: face shields, eye protection, and respirators. Overall 4261 (31%) HCWs were younger than 35 years. The majority were female participants (n=12,682, 91%). In total, 12,510 (89%) HCWs were nonimmigrant. HCW workers were distributed across 7 regions, with the largest group of 6626 (47%) working in Ontario. The majority (n=10,152, 73%) of HCWs worked full-time and 5511 (39%) were nonfrontline allied health professionals. A total of 8188 (59%) HCWs did not report exposures to confirmed or suspected COVID-19 cases. As for access to PPE, 10,758 (77%) HCWs always had access to face shields, 10,118 (72%) always had access to eye protection, and 6898 (49%) always had access to respirators.

**Table 1:** Participant demographic, occupational, and PPE^a^ access characteristics.

Characteristics			Value, n (%)
**Age (years)**			
	<35		4261 (30.5)
	35-44		3962 (28.3)
	45-54		3415 (24.4)
	≥55		2352 (16.8)
**Sex**			
	Male		1308 (9.3)
	Female		12,682 (90.7)
**Immigration status**			
	Nonimmigrant		12,510 (89.4)
	Immigrant		1480 (10.6)
**Province of workplace**			
	Atlantic provinces		1972 (14.1)
	Quebec		832 (5.9)
	Ontario		6626 (47.4)
	Manitoba and Saskatchewan		1308 (9.3)
	Alberta		2018 (14.4)
	British Columbia		1194 (8.5)
	Northern territories		40 (0.3)
**Work status**			
	Full-time		10,152 (72.6)
	Part-time		3838 (27.4)
**Frontline occupation**			
	Physician		397 (2.8)
	Nurse		4689 (33.5)
	Emergency medical personnel		223 (1.6)
**Nonfrontline occupation**			
	Personal support worker		454 (3.2)
	Allied health professional		5511 (39.4)
	Laboratory worker		1267 (9.1)
	Pharmacist		169 (1.2)
	Dental professional		1280 (9.1)
**Exposure to** **confirmed or suspected cases**			
	Yes		5802 (41.5)
	No		8188 (58.5)
**Access to PPE**			
	**Face shields**		
		Always or usually available	10,758 (76.9)
		Sometimes available	1070 (7.6)
		Never available	357 (2.6)
		Skipped answer	1805 (12.9)
	**Eye protection**		
		Always or usually available	10,118 (72.3)
		Sometimes available	1132 (8.1)
		Never available	556 (4.0)
		Skipped answer	2184 (15.6)
	**Respirators**		
		Always or usually available	6898 (49.3)
		Sometimes available	1369 (9.8)
		Never available	676 (4.8)
		Skipped answer	5047 (36.1)

^a^PPE: personal protective equipment.

### Analysis Results

In the following, we present the results for estimating the associations between the various factors and the likelihood of worsened mental health conditions for HCWs.

#### Demographic Factors

Model estimates are expressed as odds ratios (ORs), as presented in Table 2. These ORs indicate the odds of worsened mental health conditions as compared to before the start of the pandemic. Based on the ORs in Table 2, HCWs who are younger than 35 years old were more likely (OR 1.14, 95% CI 1.03-1.27; *P*=.01) to exhibit worsened mental health conditions than the reference group (35 to 44 years old). Furthermore, those aged 45-54 years and 55 years and older were less likely to exhibit worsened mental health conditions than the reference group (OR 0.71, 95% CI 0.64-0.78; *P*<.001; and OR 0.55, 95% CI 0.49-0.61; *P*<.001, respectively). Hence, the older the HCW, the lower the likelihood of worsened mental health conditions. As for sex, male HCWs were less likely (OR 0.76, 95% CI 0.67-0.86; *P*<.001) to exhibit worsened mental health conditions than their female counterparts. Immigrant HCWs were also less likely (OR 0.57, 95% CI 0.51-0.64; *P*<.001) to exhibit worsened mental health conditions than nonimmigrants. Geographically, HCWs working in Alberta have the highest likelihood of worsened mental health conditions. HCWs living in Alberta were most likely to exhibit worsened mental health conditions.

**Table 2:** Estimates for the ordinal regression model for various factors associated with the mental health conditions of HCWs^a^.

Independent variables		β	SE	OR^b^ (95% CI)	*P* value
**Age group (years; reference: 35 to 44 years)**					
	Less than 35	0.13	0.05	1.14 (1.03-1.27)	.01
	45-54	–0.35	0.05	0.71 (0.64-0.78)	<.001
	55 and older	–0.60	0.06	0.55 (0.49-0.61)	<.001
**Sex (reference: female)**					
	Male	–0.28	0.06	0.76 (0.67-0.86)	<.001
**Immigration status (reference: nonimmigrant)**					
	Immigrant	–0.56	0.06	0.57 (0.51-0.64)	<.001
**Work location (reference: Alberta)**					
	Atlantic provinces	–0.41	0.07	0.66 (0.57-0.77)	<.001
	Quebec	–0.30	0.09	0.74 (0.61-0.89)	<.001
	Manitoba and Saskatchewan	–0.09	0.08	0.91 (0.77-1.08)	.27
	Ontario	–0.24	0.06	0.78 (0.70-0.88)	<.001
	British Columbia	–0.09	0.09	0.92 (0.77-1.09)	.31
	North Territories	–0.56	0.34	0.57 (0.30-1.15)	.01
**Work status (reference: full-time)**					
	Part-time	–0.16	0.04	0.85 (0.76-0.93)	<.001
**Job setting (reference: nonfrontline)**					
	Frontline	0.23	0.05	1.26 (1.16-1.38)	<.001
**Contact with patients with COVID-19 (reference: no contact)**					
	Exposure	0.44	0.05	1.55 (1.41-1.70)	<.001
**Access to respirators (reference: did not need)**					
	Always	–0.03	0.05	0.97 (0.88-1.07)	.13
	Sometimes	0.30	0.08	1.34 (1.14-1.59)	<.001
	Never	0.27	0.10	1.31 (1.07-1.62)	<.001
**Access to eye protection (reference: did not need)**					
	Always	–0.03	0.06	0.97 (0.85-1.10)	.17
	Sometimes	0.29	0.11	1.33 (1.08-1.65)	<.001
	Never	0.41	0.13	1.51 (1.17-1.96)	<.001
**Access to face protection (reference: did not need)**					
	Always	0.26	0.07	1.30 (1.14-1.48)	<.001
	Sometimes	0.40	0.11	1.50 (1.21-1.86)	<.001
	Never	0.35	0.15	1.41 (1.05-1.92)	.02

^a^HCW: health care worker.

^b^OR: odds ratio.

#### Occupational Factors

Frontline HCWs, such as physicians, nurses, and emergency medical personnel, were more likely (OR 1.26, 95% CI 1.16-1.38; *P*<.001) to exhibit worsened mental health conditions than nonfrontline workers (personal support workers, allied health professionals, laboratory workers, pharmacists, and professionals). Part-time HCWs, however, were less likely (OR 0.85, 95% CI 0.76-0.93; *P*<.001) to exhibit worsened mental health conditions. Furthermore, HCWs who reported encountering suspected or confirmed COVID-19 cases were more likely (OR 1.55, 95% CI 1.41-1.70; *P*<.001) to exhibit worsened mental health conditions.

#### Access to PPE

HCWs who never had access to PPE such as respirators, eye protection, and face shields exhibited the highest likelihood of worsened mental health conditions than those who always had access to such PPE. For instance, HCWs who never had access to respirators were more likely (OR 1.31, 95% CI 1.07-1.62; *P*<.001) to exhibit worsened mental health conditions than those who did not need this PPE. Similar trends were exhibited with access to eye protection (OR 1.51, 95% CI 1.17-1.96; *P*<.001) and face shields (OR 1.41, 95% CI 1.05-1.92; *P*=.02).

## Discussion

### Principal Findings

The statistical analysis in this study found that HCWs who are younger than 35 years old were found to be more likely to exhibit worsened mental health conditions than HCWs aged 35-44 years. Male HCWs were less likely to exhibit worsened mental health conditions than female HCWs. Immigrant HCWs were also less likely to exhibit worsened mental health conditions than nonimmigrant HCWs. In contrast, HCWs working in Alberta had a higher likelihood of worsened mental health conditions than HCWs working elsewhere (Atlantic provinces, Quebec, Manitoba, Saskatchewan, Ontario, British Columbia, and Northern Territories). Frontline workers were more likely to exhibit worsened mental health conditions than nonfrontline HCWs. Part-time HCWs were less likely to exhibit worsened mental health conditions than full-time HCWs. HCWs who reported encountering COVID-19 cases were more likely to exhibit worsened mental health conditions than HCWs who reported no contact with the disease. As for PPE, HCWs who never had access to respirators, eye protection, and face shields were more likely to exhibit worsened mental health conditions than those who always had access to the same PPE, respectively.

### Comparison With Prior Work

Previous research has shown that shock events can result in psychological trauma to HCWs [19-21]. To the best of our knowledge, our study is the first to assess the association of a comprehensive variety of factors (demographics, occupational factors, and access to PPE) independently with the mental health conditions of HCWs.

With regard to demographic factors, a study found sex to be associated with worsened mental health conditions of HCWs. Female medical staff exhibited a higher incidence of severe anxiety than their male counterparts [22]. Sex was also found as a predictor of increased anxiety and distress in a study that found female HCWs more vulnerable to such conditions than male HCWs [23]. Our study findings corroborate these previous findings. Similarly, for age, our results demonstrated that older HCWs exhibited less likelihood of worsened mental health conditions. This agrees with findings from a study in the Middle East that found older workers enjoyed better mental health conditions than younger workers [24]. Older HCWs with longer work experience seemed to have handled the pandemic better than their younger counterparts. Another study found that HCWs 40 years or older were less likely to report higher anxiety during the pandemic than younger HCWs [25]. Our results confirm such findings as well.

As for occupational factors, our findings align with a cross-sectional study conducted in Oman, which found frontline workers to be more likely to have anxiety and sleep problems [26]. This was attributed to frontline workers’ increased awareness of the mortality rate of COVID-19 and their fear of contracting the virus. Similar findings were reported by a study conducted in China as well [27]. Exposure to COVID-19 cases has been recognized as a risk factor associated with an increased likelihood of mental health issues in the literature. For instance, in France, female urologists working on the frontline were 1.41 times more likely to feel a degree of stress during their duties. Of this group, those who worked in a department where patients with COVID-19 are treated were 1.85 times more likely to report a degree of stress during work duties [28]. This was attributed to the workers’ fear of infection and the spread of the virus. Direct involvement with COVID-19 care is also found to be highly associated with fear, depression, and anxiety as compared with those working under lower risk conditions [29]. The authors attributed this to the workers’ fear of bringing the virus to their families at home.

As for the work setting, contrary to our findings, the literature reports higher levels of fear and anxiety among part-time HCWs than among full-time [30]. Another study in France found part-time HCWs to exhibit a greater association with distress [31]. Given the small number of part-time HCWs in our study, caution should be observed in interpreting the results. Regarding factors related to access to PPE, access to PPE was found to be associated with better health and less stress [24]. Lack of access to PPE was found to be a major source of HCW stress in a study that surveyed emergency physicians [32]. In Canada, a study found that inadequate PPE supply is associated with increased symptoms of anxiety and depression among HCWs [33]. Workers were concerned about the ability to access sufficient PPE during work hours. These findings are in line with our results. Compared to studies in the literature, our study is the first comprehensive study that assesses the association of a diverse pool of factors including demographics, occupational, and access to PPE, with HCWs’ mental health conditions.

### Implications: Mental Health Programming

Our comprehensive study sheds light on the association of various factors with HCWs’ mental health after the start of the pandemic. It helps in understanding the vulnerability of various HCW groups to mental health during such events. Certain groups were at substantially higher risk of exhibiting worsened mental health conditions after the pandemic, hence the need for a specialized support program to target this group. As a direct implication, the findings can be used to inform guidelines for mental health support for HCWs during future public health emergencies. Such mental health support may be directed more specifically to more vulnerable groups. Literature has pointed to the importance of an evidence-based approach to designing mental health support programs for HCWs [34,35]. For instance, a study in Alberta during the pandemic pointed to the importance of understanding HCWs’ occupational settings and mental health mitigation techniques [16]. Others have discussed the importance of support programs for HCWs during the pandemic based on need and background [36,37]. More importantly, many studies highlighted the importance of designing personalized mental health support programs based on various factors including demographic and occupational factors [38,39]. Some of these programs used mobile technology to offer mental health support to HCWs [35,40]. As such, our study aims to inform stakeholders of such factors associated with HCW groups most vulnerable to future events such as the pandemic and health crises.

### Limitations and Future Directions

This study has several limitations. First, the scope of the study was limited to assessing mental health in general without measuring different mental condition forms such as depression, anxiety, and fear. This is due to the limited data offered by the survey we used. Second, participants were asked, “Compared to before the COVID-19 pandemic, how would you say your mental health is now?” That is, HCWs had to objectively compare their mental health state from the time they received the survey to sometime in the past. There may be some variation in how HCWs perceive this comparison. Third, data collected were at a single point in time, which limits the ability to compare mental health assessments to later periods in the pandemic and during subsequent pandemic waves. Fourth, no information about HCW medical history is collected in this survey. In future research, we recommend collecting data at different points in the given event to allow for a time-series analysis and comparison at different crucial points of the event. We also suggest the collection of distress information and whether the conditions persisted beyond the initial shockwave. HCWs’ years of work experience and ethnicity information can also be useful in future studies. We also believe medical history, particularly a preexisting history of mental health disorders, is an important factor associated with the mental health conditions of HCWs and should be considered in future studies.

### Conclusions

This study investigated the factors associated with worsened mental health conditions of HCWs in Canada during the pandemic. Our study suggested the association of various factors with the likelihood of HCWs exhibiting worsened mental health conditions as compared to feeling neutral and better. In agreement with the literature, our findings concluded that younger (vs older), female (vs male), nonimmigrant (vs immigrant), full-time (vs part-time), and frontline (vs nonfrontline) HCWs living in Alberta (vs other provinces) exhibited a higher likelihood of worsened mental health conditions than those who felt neutral or better. Those who reported concerns about access to PPE also exhibited the same trend. Such findings can guide the future development of health care programming and inform mental health support planning for HCWs. COVID-19 is a shocking event that introduced uncertainty to the health care system.

## Appendix

Multimedia Appendix 1 Logistic model.

## Abbreviations

HCW: health care worker
OR: odds ratio
PPE: personal protective equipment

## Data Availability

The data that support the findings of this study is available through Statistics Canada.

## References

[1] (2020). WHO Director-General's opening remarks at the media briefing on COVID-19—11 March 2020. World Health Organization.

[2] (2021). Overview: COVID-19's impact on health care systems. Canadian Institute for Health Information.

[3] Dudevich A, Frood J (2021). Impact of the COVID-19 pandemic on health system use in Canada. Healthc Q.

[4] Carazo S, Pelletier M, Talbot D, Jauvin N, De Serres G, Vézina M (2022). Psychological distress of healthcare workers in Québec (Canada) during the second and the third pandemic waves. J Occup Environ Med.

[5] Brophy JT, Keith MM, Hurley M, McArthur JE (2021). Sacrificed: Ontario healthcare workers in the time of COVID-19. New Solut.

[6] Stuijfzand S, Deforges C, Sandoz V, Sajin CT, Jaques C, Elmers J, Horsch A (2020). Psychological impact of an epidemic/pandemic on the mental health of healthcare professionals: a rapid review. BMC Public Health.

[7] Vizheh M, Qorbani M, Arzaghi SM, Muhidin S, Javanmard Z, Esmaeili M (2020). The mental health of healthcare workers in the COVID-19 pandemic: a systematic review. J Diabetes Metab Disord.

[8] Liu JJW, Nazarov A, Plouffe RA, Forchuk CA, Deda E, Gargala D, Le T, Bourret-Gheysen J, Soares V, Nouri MS, Hosseiny F, Smith P, Roth M, MacDougall AG, Marlborough M, Jetly R, Heber A, Albuquerque J, Lanius R, Balderson K, Dupuis G, Mehta V, Richardson JD (2021). Exploring the well-being of health care workers during the COVID-19 pandemic: protocol for a prospective longitudinal study. JMIR Res Protoc.

[9] Cai H, Tu B, Ma J, Chen L, Fu L, Jiang Y, Zhuang Q (2020). Psychological impact and coping strategies of frontline medical staff in hunan between january and march 2020 during the outbreak of coronavirus disease 2019 (COVID-19) in Hubei, China. Med Sci Monit.

[10] Peeri NC, Shrestha N, Rahman MS, Zaki R, Tan Z, Bibi S, Baghbanzadeh M, Aghamohammadi N, Zhang W, Haque U (2020). The SARS, MERS and novel coronavirus (COVID-19) epidemics, the newest and biggest global health threats: what lessons have we learned?. Int J Epidemiol.

[11] De Brier N, Stroobants S, Vandekerckhove P, De Buck E (2020). Factors affecting mental health of health care workers during coronavirus disease outbreaks (SARS, MERS and COVID-19): a rapid systematic review. PLoS One.

[12] Badahdah A, Khamis F, Al Mahyijari N, Al Balushi M, Al Hatmi H, Al Salmi I, Albulushi Z, Al Noomani J (2021). The mental health of health care workers in Oman during the COVID-19 pandemic. Int J Soc Psychiatry.

[13] Yıldırım M, Arslan G, Özaslan A (2022). Perceived risk and mental health problems among healthcare professionals during COVID-19 pandemic: exploring the mediating effects of resilience and coronavirus fear. Int J Ment Health Addict.

[14] Serrano-Ripoll MJ, Meneses-Echavez JF, Ricci-Cabello I, Fraile-Navarro D, Fiol-deRoque MA, Pastor-Moreno G, Castro A, Ruiz-Pérez I, Zamanillo Campos R, Gonçalves-Bradley DC (2020). Impact of viral epidemic outbreaks on mental health of healthcare workers: a rapid systematic review and meta-analysis. J Affect Disord.

[15] Gupta B, Sharma V, Kumar N, Mahajan A (2020). Anxiety and sleep disturbances among health care workers during the COVID-19 pandemic in India: cross-sectional online survey. JMIR Public Health Surveill.

[16] Mrklas K, Shalaby R, Hrabok M, Gusnowski A, Vuong W, Surood S, Urichuk L, Li D, Li XM, Greenshaw AJ, Agyapong VIO (2020). Prevalence of perceived stress, anxiety, depression, and obsessive-compulsive symptoms in health care workers and other workers in Alberta during the COVID-19 pandemic: cross-sectional survey. JMIR Ment Health.

[17] Hadley J, Waidmann T (2006). Health insurance and health at age 65: implications for medical care spending on new Medicare beneficiaries. Health Serv Res.

[18] Yunchao C, Yusof SA, Amin RM, Arshad MNM (2020). Household debt and household spending behavior: evidence from Malaysia. J Ekon Malays.

[19] Lancee WJ, Maunder RG, Goldbloom DS, Coauthors for the Impact of SARS Study (2008). Prevalence of psychiatric disorders among Toronto hospital workers one to two years after the SARS outbreak. Psychiatr Serv.

[20] Maunder RG, Lancee WJ, Balderson KE, Bennett JP, Borgundvaag B, Evans S, Fernandes CMB, Goldbloom DS, Gupta M, Hunter JJ, McGillis Hall L, Nagle LM, Pain C, Peczeniuk SS, Raymond G, Read N, Rourke SB, Steinberg RJ, Stewart TE, VanDeVelde-Coke S, Veldhorst GG, Wasylenki DA (2006). Long-term psychological and occupational effects of providing hospital healthcare during SARS outbreak. Emerg Infect Dis.

[21] Park J, Lee E, Park N, Choi YH (2018). Mental health of nurses working at a government-designated hospital during a MERS-CoV outbreak: a cross-sectional study. Arch Psychiatr Nurs.

[22] Huang JZ, Han MF, Luo TD, Ren AK, Zhou XP (2020). [Mental health survey of medical staff in a tertiary infectious disease hospital for COVID-19]. Zhonghua Lao Dong Wei Sheng Zhi Ye Bing Za Zhi.

[23] Lai J, Ma S, Wang Y, Cai Z, Hu J, Wei N, Wu J, Du H, Chen T, Li R, Tan H, Kang L, Yao L, Huang M, Wang H, Wang G, Liu Z, Hu S (2020). Factors associated with mental health outcomes among health care workers exposed to coronavirus disease 2019. JAMA Netw Open.

[24] Zhang SX, Liu J, Jahanshahi AA, Nawaser K, Yousefi A, Li J, Sun S (2020). At the height of the storm: healthcare staff's health conditions and job satisfaction and their associated predictors during the epidemic peak of COVID-19. Brain Behav Immun.

[25] Van Wert MJ, Gandhi S, Gupta I, Singh A, Eid SM, Haroon Burhanullah M, Michtalik H, Malik M (2022). Healthcare worker mental health after the initial peak of the COVID-19 pandemic: a US Medical Center cross-sectional survey. J Gen Intern Med.

[26] Alshekaili M, Hassan W, Al Said N, Al Sulaimani F, Jayapal SK, Al-Mawali A, Chan MF, Mahadevan S, Al-Adawi S (2020). Factors associated with mental health outcomes across healthcare settings in Oman during COVID-19: frontline versus non-frontline healthcare workers. BMJ Open.

[27] Cai Q, Feng H, Huang J, Wang M, Wang Q, Lu X, Xie Y, Wang X, Liu Z, Hou B, Ouyang K, Pan J, Li Q, Fu B, Deng Y, Liu Y (2020). The mental health of frontline and non-frontline medical workers during the coronavirus disease 2019 (COVID-19) outbreak in China: a case-control study. J Affect Disord.

[28] Abdessater M, Rouprêt M, Misrai V, Matillon X, Gondran-Tellier B, Freton L, Vallée M, Dominique I, Felber M, Khene ZE, Fortier E, Lannes F, Michiels C, Grevez T, Szabla N, Boustany J, Bardet F, Kaulanjan K, de Mazancourt ES, Ploussard G, Pinar U, Pradere B (2020). COVID19 pandemic impacts on anxiety of French urologist in training: outcomes from a national survey. Prog Urol.

[29] Lu W, Wang H, Lin Y, Li L (2020). Psychological status of medical workforce during the COVID-19 pandemic: a cross-sectional study. Psychiatry Res.

[30] Labrague LJ, de Los Santos JAA (2021). Fear of COVID-19, psychological distress, work satisfaction and turnover intention among frontline nurses. J Nurs Manag.

[31] Fournier A, Laurent A, Lheureux F, Ribeiro-Marthoud MA, Ecarnot F, Binquet C, Quenot J (2022). Impact of the COVID-19 pandemic on the mental health of professionals in 77 hospitals in France. PLoS One.

[32] Marco CA, Larkin GL, Feeser VR, Monti JE, Vearrier L, ACEP Ethics Committee (2020). Post-traumatic stress and stress disorders during the COVID-19 pandemic: survey of emergency physicians. J Am Coll Emerg Physicians Open.

[33] Oudyk J, Smith P (2020). Occupational Health Clinics for Ontario workers mayday, mayday workplace mental health series 2020. Occupational Health Clinics for Ontario.

[34] Greenberg N (2020). Mental health of health-care workers in the COVID-19 era. Nat Rev Nephrol.

[35] Lewis M, Palmer VJ, Kotevski A, Densley K, O'Donnell ML, Johnson C, Wohlgezogen F, Gray K, Robins-Browne K, Burchill L (2021). Rapid design and delivery of an experience-based co-designed mobile app to support the mental health needs of health care workers affected by the COVID-19 pandemic: impact evaluation protocol. JMIR Res Protoc.

[36] Vizheh M, Qorbani M, Arzaghi SM, Muhidin S, Javanmard Z, Esmaeili M (2020). The mental health of healthcare workers in the COVID-19 pandemic: a systematic review. J Diabetes Metab Disord.

[37] David E, DePierro JM, Marin DB, Sharma V, Charney DS, Katz CL (2022). COVID-19 pandemic support programs for healthcare workers and implications for occupational mental health: a narrative review. Psychiatr Q.

[38] Siskind D, Harris M, Pirkis J, Whiteford H (2012). Personalised support delivered by support workers for people with severe and persistent mental illness: a systematic review of patient outcomes. Epidemiol Psychiatr Sci.

[39] Deif R, Salama M (2021). Depression from a precision mental health perspective: utilizing personalized conceptualizations to guide personalized treatments. Front Psychiatry.

[40] Fiol-DeRoque MA, Serrano-Ripoll MJ, Jiménez R, Zamanillo-Campos R, Yáñez-Juan AM, Bennasar-Veny M, Leiva A, Gervilla E, García-Buades ME, García-Toro M, Alonso-Coello P, Pastor-Moreno G, Ruiz-Pérez I, Sitges C, García-Campayo J, Llobera-Cánaves J, Ricci-Cabello I (2021). A mobile phone-based intervention to reduce mental health problems in health care workers during the COVID-19 pandemic (PsyCovidApp): randomized controlled trial. JMIR Mhealth Uhealth.

