# Supplementary material for: Factors Associated With Worsened Mental Health of Health Care Workers in Canada During the COVID-19 Pandemic: Cross-Sectional Survey Study
Source: Interact J Med Res. 2024 Feb 15;13:e50064. doi: 10.2196/50064 (PMC10905361; doi:10.2196/50064)
Supplement: Multimedia Appendix 1 [file ijmr_v13i1e50064_app1.docx]

**Table of contents**

[Statistical details of the modelling approach 2](#_Toc103588833)

# **Statistical details of the modeling approach**

We employed an ordinal logistic regression model to control the impact of each factor as an independent variable separately. Those factors include demographics, occupational, and access levels to PPEs. The model is given in the following general form:

$$\boldsymbol{ln}\left( \boldsymbol{Prob}\left( S \leq j \right) \right)=\alpha_{n} - \left[ \beta_{1}X_{1}+ \beta_{2}X_{2}+\ldots+ \beta_{23}X_{23} \right]$$

This model estimated the probability that the state S is equal to j. Here, j = 1 refers to better mental health conditions for the HCW, j = 2 refers to the same mental health conditions for the HCW, and j = 3 refers to worse mental health conditions for the HCW, all compared to before the start of the pandemic. Further, $\alpha_{n}$refers to the cut-off points, where n= 1,...,j-1.
